# Influence of symmetry breaking degrees on surface plasmon polaritons propagation in branched silver nanowire waveguides

**DOI:** 10.1038/srep34418

**Published:** 2016-09-28

**Authors:** Jiaojiao Hua, Fan Wu, Zhongfeng Xu, Wenhui Wang

**Affiliations:** 1 School of science, Xi’an Jiaotong University, Xi’an, 710049, China

## Abstract

Surface plasmon polaritons (SPPs)-based nanowire (NW) waveguides demonstrate promising potentials in the integrated nanophotonic circuits and devices. The realization of controlling SPPs propagation in NWs is significant for the performance of nanophotonic devices when employed for special function. In this work, we report the effect of symmetry breaking degrees on SPPs propagation behavior in manually fabricated branched silver NW structures. The symmetry breaking degree can be tuned by the angle between main NW and branch NW, which influences the emissions at the junction and the main NW terminal in a large extent. Our results illustrate the significance of symmetry breaking degree on SPPs propagation in NW-based waveguides which is crucial for designing the future nanophotonic circuits.

Manipulation of light at subwavelength scale is crucial for the development of highly integrated nanophotonic devices^1^,^2^. Due to their unique ability to concentrate light into nanoscale regions^3^,^4^,^5^,^6^,^7^, surface plasmon polaritons (SPPs) which are the collective oscillation of free electrons with electromagnetic waves at the metal/dielectric interface^8^ have drawn increasing interest at various fields, such as super-resolution imaging^9^,^10^, nanolaser^11^,^12^, nanoantenna^13^,^14^ and resonators^15^,^16^. Due to the unique feature of nanowire (NW) used in the integrated circuits and devices^17^, SPPs-based NW waveguides show potential applications for realizing special nanophotonic components, such as logic gates^18^,^19^, switchers^20^,^21^, multiplexers and routers^22^,^23^. The routing and logic functions rely on the controllable SPPs propagation in the NWs. Consequently, the control of SPPs propagation and the position at which SPPs can be selectively scattered into photons are significant for the development of nanophotonic devices. As is well known, the momentum of SPPs is larger than that of photon at the free space^5^,^24^,^25^. Therefore, free photons cannot directly excite SPPs, and SPPs cannot be directly scattered from NW either. In order to compensate the mismatch of the momentum, the introduction of symmetry breaking is an efficient and feasible way.

In general, the symmetry breaking in NW structures can be introduced by three methods: bending the NW^26^,^27^,^28^, placing a nanoparticle (NP)^29^,^30^,^31^ or introducing another NW^22^,^28^,^32^. Specifically, when a NW is bent, the structural symmetry is broken and SPPs can be scattered into photons in the bent area. Through continuously bending the NW, Wang *et al.*^26^ investigated the relationship between the propagation loss and bending radius, which showed that the energy loss is increased with decreasing the bending radius. In addition, NP can also be employed to break the structural symmetry in NW. Sun *et al.*^33^,^34^,^35^ reported that the SPPs can be scattered into photons at the position of NP, which can be used for the remote excitation in the nanophotonic circuits. Similarly, the introduction of another NW also causes the symmetry breaking at the position of the junction in branched NW structures. Fang *et al.*^22^ reported that the SPPs can be routed into different wire branches and thus the branched structures can be used as plasmonic multiplexers and routers. Nevertheless, little research work has been done on the influence of the structural symmetry breaking degrees, which have a non-negligible effect on controlling SPPs propagation in the NW network to realize some special advanced functions.

In this work, we investigate the effect of symmetry breaking degrees on SPPs propagation properties by introducing different angles in branched silver NW structures. SPPs can be scattered into free photons at the position where symmetry breaking occurs, such as junction and NW terminals. The symmetry breaking degrees are changed manually through precisely tuning the branch angles in our experiments. We find that the emission intensities at the junction and main NW end can be affected by the branch angles. The emission intensity at junction is nonlinearly changed with the branch angle, that is, the intensity is nonlinearly varied with the symmetry breaking degree. It is interesting to note that there exists a minimum value in the emission intensity when changing symmetry breaking degree. Our experimental results give a preliminary relationship between symmetry breaking degree and emission intensity in SPPs-based NW waveguides, which is instructive to the design and fabrication of future nanophotonic circuits and devices.

## Experimental Methods

The Ag NWs were synthesized through a multi-step polyol process method^36^. The ethanol suspension of Ag NWs was deposited on a clean glass substrate and dried under ambient condition. The branched NW structure was manually fabricated using a tapered optical fiber controlled by a 3D moving stage under an inverted optical microscope (IX73, Olympus). In addition, another tapered optical fiber was employed to excite SPPs in the main NW. The emission intensities at the NW terminals and the junction were recorded by a 50 × objective (N.A. = 0.80, Olympus) and CCD camera (1500-GE-TE, Thorlabs). In the experiment, a laser (DL785-070-SO, CrystaLaser) of 785 nm emitting wavelength was used for SPP excitation. The scanning electron microscope (SEM) images of branched structures were taken on a field-emission microscope (JEOL JSM-7000F) operated at an accelerating voltage of 15 kV.

## Results and Discussions

**Figure 1:**
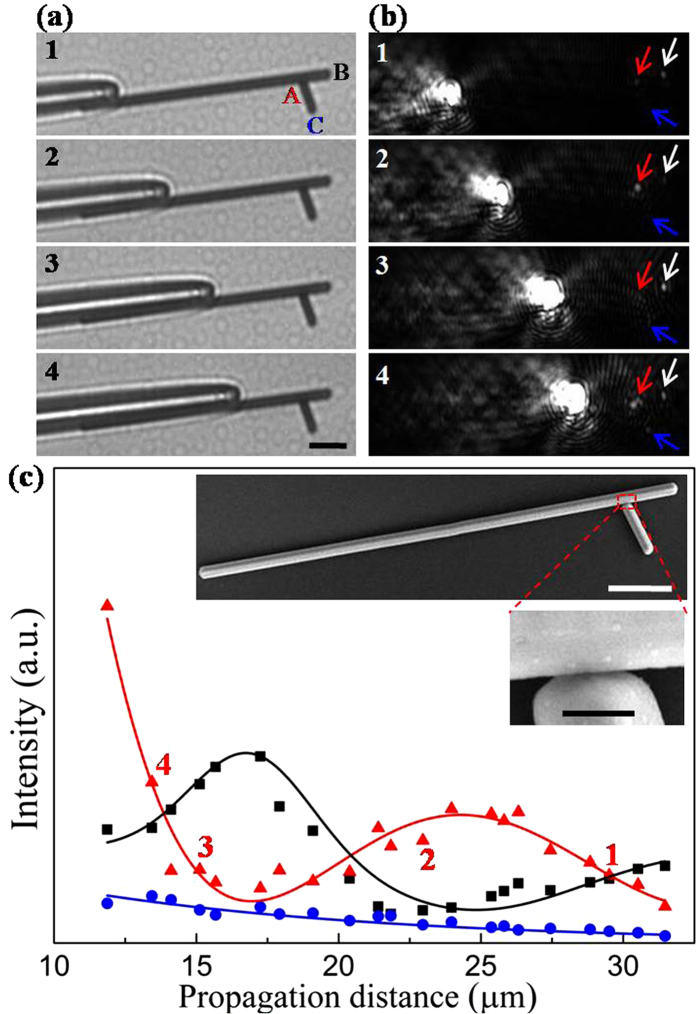
Characterization of propagation in the branched NW structure. (**a**) Selected bright-field optical images and (**b**) corresponding dark-field optical images for different propagation distances. The emission spots from junction, main NW end and branch NW end are indicated by the corresponding arrows. The scale bar is 5 μm. (**c**) The output intensity at junction A (red triangles), main NW end B (black squares) and branch NW end C (blue circles) for varying propagation distances. The solid curves are the corresponding fitted output intensities. The red triangles marked by numerals are corresponding values of emission light for (**b**). The insets in (**c**) show the SEM images of the branched NW structure. The scale bars are 5 μm (top) and 500 nm (bottom), respectively. The angle between the main NW and the branch NW is approximately 75°. The length and diameter of the main NW are 35.7 μm and 920 nm, and the length and diameter of branch NW are 4.1 μm and 800 nm. The distance from the junction to main NW end is about 3.7 μm.

The propagation properties of SPPs in the branched NW structure with the angle about 75° are shown in Fig. 1. In these experiments, the propagation distance is defined as the distance from the excitation end to the main NW terminal. Through continuously moving the excitation position, the propagation distance is decreased, and the emission intensities at the junction, main NW end and branch NW end can also be changed accordingly, which are shown in Fig. 1(b). For branched NW structure, SPPs could be scattered into photons not only at the main NW end but also at the junction. As a result, emission spots from junction and NW terminals can be seen from the dark-field optical images, as displayed in Fig. 1(b). Figure 1(a) displays the corresponding bright-field optical images. Figure 1(c) shows the quantitative description of the emissions in branched NW structure. It can be clearly seen in Fig. 1(c) that the output intensity at the junction exhibits exponential attenuation plus an approximate sine oscillation when the propagation distance increases, whereas the emission intensity at main NW end only present a sine-like oscillation. In addition, the emitted light at branch NW end is rather feeble. These results indicate that the junction plays a dominant role in controlling the propagation behavior of SPPs in the branched NWs and a large proportion of SPPs are scattered into photons at the junction. The exponential damping trend of the junction behaves like the case in the individual NW. As is well known, the exponential attenuation of the emission at the end of an individual NW arises from the Ohmic loss in NW^37^,^38^. It is reasonable to infer that the exponential damping at the junction arises from the intrinsic Ohmic loss in the main NW. In addition, it is interesting to note that the emission intensity at the junction shows a damping oscillating tendency with increasing the propagation distance larger than 15 μm (Fig. 1(c)). This can be probably attributed to the local SPPs field distribution in the main NW. In general, the field distribution caused by the superposition of different plasmon modes presents the attenuating period oscillation pattern on the NW^39^. The antinode of near SPPs field distribution is defined as the position with larger field intensity, and the node is the position with weaker intensity in NW. When the branch NW is placed properly so that the junction is just on the antinode, more proportion of SPPs in main NW can be scattered into photons, and *vice versa*. With the propagation distance continuously changed, the position of junction alternately goes through the node and antinode, thus, the emission intensity at junction shows a damping sine-like variation.

For the emission at main NW terminal, it is found that the emission intensity shows an opposite oscillation towards the case in the junction (Fig. 1(c)). More concretely, when the propagation distance is 16.8 μm, the output intensity at the junction is weak while the emission intensity at main NW end is strong. In contrast, at the propagation distance of 24.5 μm, the value is reversed. Similarly, this can be probably ascribed to the local SPPs field distribution in the main NW. The junction is probably located in the antinode at the propagation distance of 24.5 μm. More proportion of SPPs is scattered into photons resulting in the large emission intensity at the junction, while only a small proportion of SPPs is left in the main NW resulting in the weak output intensity at main NW end. In addition, it is noted that the period of the oscillation for emission intensity at junction and main NW end is about 7 μm, which can roughly reflect the period of standing wave distribution along the main wire. Compared with the previous report^18^, this period is longer, which probably originates from the complicated SPP modes and decreased energy loss in the thick NW. Different from the emitted intensities at junction and main NW end in this manually fabricated branched structure, the emission intensity at branch NW end is faint (as vividly displayed in Fig. 1(c)). It is very different from the case which has shown good performance in the branch NW emission as reported^22^,^40^. In their experiments, the branched NW structures are occasionally formed. The weak emission at branch NW end can be probably ascribed to the low efficiency of SPPs coupled into the branch wire in our experiment, resulting from the loose contact at the junction in the manually fabricated branched structures.

**Figure 2:**
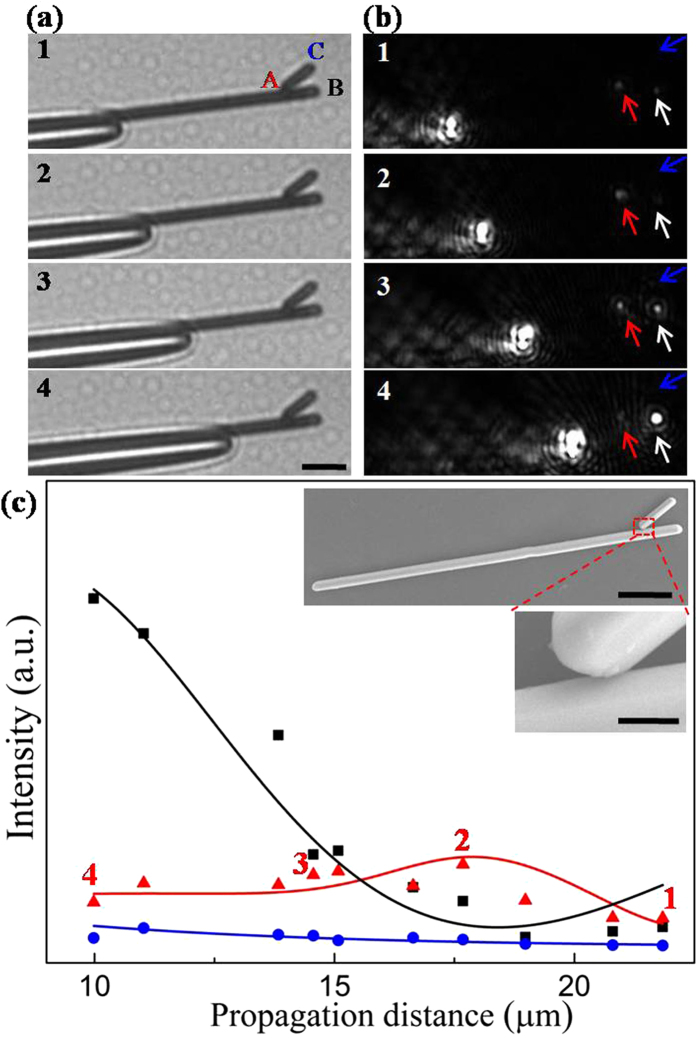
Characterization of propagation in the branched NW structure. (**a**) Selected bright-field optical images and (**b**) corresponding dark-field optical images for different propagation distances. The emission spots from junction and NW terminals are indicated by the corresponding arrows. The scale bar is 5 μm. (**c**) The output intensity at junction A (red triangles), NW end B (black squares) and C (blue circles) for varying propagation distances. The solid curves are the corresponding fitted output intensity. The red triangles marked by numerals are corresponding values of emission light for (**b**). The insets in (**c**) show the SEM images of the branched NW structure. The scale bars are 5 μm (top) and 500 nm (bottom), respectively. The angle between two NWs is about 32°. The length and diameter of the main NW are 34.9 μm and 980 nm, and that of branch NW are 4.2 μm and 820 nm. The distance from the junction to main NW end is about 3.6 μm.

In some experiments, like the case shown in Fig. 2, we find that the angle between main NW and branch NW has a major effect on the propagation behavior of SPPs in branched NW structure. These results are greatly different from the situation in Fig. 1. The angle in the branched NW structure is about 32°. It can be seen in Fig. 2(c) that, the emission at main NW end shows an exponential damping trend with a fluctuation, while the emission intensity at the junction displays an attenuating oscillation which is opposite to that of main NW end. Compared with the structure in Fig. 1, it is found that the parameters of the nanostructure in Fig. 2, such as the distance between junction and main NW end, the size of main and branch NW are all almost the same except the different angles in these two branched structures. Therefore, it is reasonable to infer that the difference of the propagation behaviors of SPPs in these two branched structures probably originates from the effect of the branch angle. The change of angle causes the variation of symmetry breaking degree which further affects the SPPs propagation in the branched NW structure. Consequently, the different symmetry breaking degrees cause the variation of propagation behaviors as shown in Figs 1 and 2.

**Figure 3:**
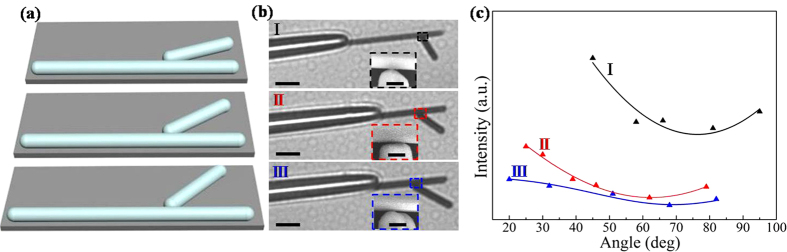
(**a**) Schematic diagram of the change of angle in an individual branched NW structure. (**b**) Selected bright-field optical images for three different branched structures (I, II, and III). The scale bar is 5 μm. The insets are corresponding SEM images of the junction section. The scale bar is 500 nm. (**c**) The output intensity at junction (triangles) and corresponding fitted output intensity (solid curves).

In order to further investigate the effect of the symmetry breaking degrees on the propagation properties of SPPs in branched NW structures, we performed a series of experiments through continuously changing the angle in the same branched NW structure. The variation of angle leads to different symmetry breaking degree. In these experiments, only the angle is changed while other experiment conditions are kept the same, as shown in Fig. 3(a). During the process of carefully changing the angle, the distance between branch NW end and main NW does not have an obvious change, and the change of contact area should be very small. Compared with the effect of branch angle, contact area has less impact on the emission behavior. As the emission intensity at junction can directly reflect the effect of symmetry breaking degree, intensity change at junction has been used to study the influence of branch angle on SPPs propagation behavior. Figure 3(b) shows three selected branched structures. Figure 3(c) displays the change of intensities at junctions of the three different branched NW structures with the branch angles. It can be seen from the Fig. 3(c) that the emitted intensities at the junctions in structure I, II, and III vary nonlinearly with the branch angles rather than the monotonic increasing tendencies as we expected. There are minimum values exist in the intensities at junctions when changing the angles, in other words, the emission intensity at junction varies nonlinearly with the change of symmetry breaking degree. In addition, it is worth to note that the corresponding minimum values of emission intensities at junctions are not the same in these three branched structures, as well as the overall emission intensities at the junctions. These should be attributed to the difference of morphology of branch NW end. Although the morphology of branch NW end probably affects the specific minimum value when symmetry breaking degree is changed, the relationship between the symmetry breaking degree and emission intensity at the junction shows the same trend (nonlinearity with a minimum value) for various branched NW structures in our experiments. It is an interesting phenomenon, and the research on the deep understanding of underlying mechanism is in progress.

**Figure 4:**
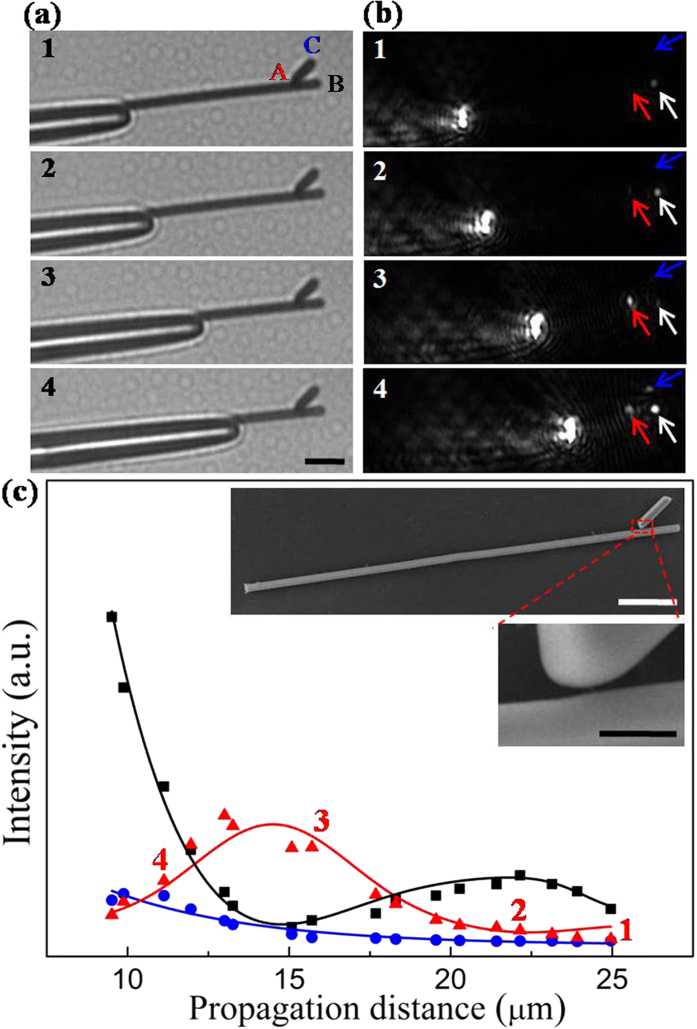
(**a**) Selected bright-field optical images and (**b**) corresponding dark-field optical images for different propagation distances. The emission spots from junction and NW terminals are indicated by the corresponding arrows. The scale bar is 5 μm. (**c**) The output intensity at junction A, main NW end B and branch NW end C, and the corresponding fitted curves for varying propagation distances. The red triangles marked by numerals are corresponding values of emission light for (**b**). Insets are SEM images of the branched NW structure. The scale bars are 5 μm (top) and 500 nm (bottom), respectively. The angle between two NWs is approximately 37°. The length and diameter of the main NW are 37.6 μm and 640 nm, and the length and diameter of the branch NW 3.6 μm and 790 nm. The distance from the junction to main NW end is about 3.2 μm.

It should be pointed out that there is no observable gap between the main NW and branch NW in previous branched structures as the gap cannot be easily seen in the SEM images (the gap may be only few nanometers which cannot be identified by SEM). Among these numerous branched NW structures measured for the SPPs propagation properties in our experiments, we find that some structures have small gap, like the case shown in Fig. 4. The gap is about 40 nm. As shown in Fig. 4(c), when the propagation distance increases, the emission intensity at main NW end exponentially decreases plus a sine-like oscillation, while the emission at the junction shows a sine-like oscillation which is opposite to that of main NW end. The period of the oscillation for emission intensity is about 7 μm. Meanwhile, the emission intensity at branch NW end is still weak. The SPPs propagating behavior is almost the same as the situation in Fig. 2. Compared with variation on the morphology (shape and size) of these two branched structures, the change of angle has a major influence on the emission behavior. As the branch angle is similar to the case in Fig. 2, it is reasonable to deduce that the existence of small gap can rarely affect the primary propagation properties of SPPs in branched NW structure. Thus, this gap should not have effective influence on the behavior of emission at the junction when the angle is changed in branched NW structure at Fig. 3.

## Conclusion

The effect of symmetry breaking degrees on SPPs propagation properties in manually fabricated branched NW structures has been investigated. Our experimental results demonstrate that the propagation properties of SPPs can be greatly affected by the angle between main NW and branch NW. Different angles correspond to different symmetry breaking degrees. When the symmetry breaking degrees are changed, SPPs show different propagation behaviors. In some branched NW structures, junction plays a major role in controlling SPPs propagation behavior rather than main NW end. Therefore, the emission intensity at junction presents exponential decay trend plus a damping sine-like oscillation, while the intensity at main NW end only shows an opposite sine-like oscillation tendency. In other branched structures, the situation is reversed. By investigating the effect of branch angle on emission intensity at the junction, we find that the relationship between emission intensity at junction and symmetry breaking degree is nonlinear, and there exists a minimum value in the emission intensity. In addition, the existence of the small gap between the main NW and branch NW rarely influence the primary propagation properties of SPPs in branched structure. Our research on the effect of symmetry breaking degrees in SPPs-based NW waveguides shed light on the future design of nanophotonic circuits and devices.

## Additional Information

**How to cite this article**: Hua, J. *et al.* Influence of symmetry breaking degrees on surface plasmon polaritons propagation in branched silver nanowire waveguides. *Sci. Rep.* **6**, 34418; doi: 10.1038/srep34418 (2016).
